# Angiopoietin‐2 reverses endothelial cell dysfunction in progeria vasculature

**DOI:** 10.1111/acel.14375

**Published:** 2024-10-18

**Authors:** Sahar Vakili, Elizabeth K. Izydore, Leonhard Losert, Wayne A. Cabral, Urraca L. Tavarez, Kevin Shores, Huijing Xue, Michael R. Erdos, George A. Truskey, Francis S. Collins, Kan Cao

**Affiliations:** ^1^ Department of Cell Biology and Molecular Genetics University of Maryland College Park Maryland USA; ^2^ Molecular Genetics Section, Center for Precision Health Research, National Human Genome Research Institute National Institutes of Health Bethesda Maryland USA; ^3^ Department of Biomedical Engineering Duke University Durham North Carolina USA; ^4^ Frederick National Laboratory for Cancer Research Frederick Maryland USA

**Keywords:** aging, Angiopoietin‐2, CVD, endothelial cells dysfunction, HGPS

## Abstract

Hutchinson‐Gilford progeria syndrome (HGPS) is a rare premature aging disorder in children caused by a point mutation in the lamin A gene, resulting in a toxic form of lamin A called progerin. Accelerated atherosclerosis leading to heart attack and stroke are the major causes of death in these patients. Endothelial cell (EC) dysfunction contributes to the pathogenesis of HGPS related cardiovascular diseases (CVD). Endothelial cell–cell communications are important in the development of the vasculature, and their disruptions contribute to cardiovascular pathology. However, it is unclear how progerin interferes with such communications that lead to vascular dysfunction. An antibody array screening of healthy and HGPS patient EC secretomes identified Angiopoietin‐2 (Ang2) as a down‐regulated signaling molecule in HGPS ECs. A similar down‐regulation of *Ang2* mRNA and protein was detected in the aortas from an HGPS mouse model. Addition of Ang2 to HGPS ECs rescues vasculogenesis, normalizes endothelial cell migration and gene expression, and restores nitric oxide bioavailability through eNOS activation. Furthermore, Ang2 addition reverses unfavorable paracrine effects of HGPS ECs on vascular smooth muscle cells. Lastly, by utilizing adenine base editor (ABE)‐corrected HGPS ECs and progerin‐expressing HUVECs, we demonstrated a negative correlation between progerin and Ang2 expression. Lastly, our results indicated that Ang2 exerts its beneficial effect in ECs through Tie2 receptor binding, activating an Akt‐mediated pathway. Together, these results provide molecular insights into EC dysfunction in HGPS and suggest that Ang2 treatment has potential therapeutic effects in HGPS‐related CVD.

## Graphical abstract

Progerin expression in the nucleus causes downregulation of Ang2, which causes defects in HGPS ECs tube formation and nitric oxide production. Ang2 treatment rescues HGPS ECs angiogenesis, migration, nitric oxide production and most importantly paracrine signaling.

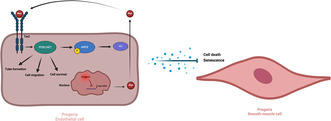

**Abbreviations**
ABE: adenine base editor
Ang2: angiopoietin‐2
C12FDG: C12‐fluorescein di‐β‐D‐galactopyranoside
CM: conditioned medium
CRISPR: clustered regularly interspaced short palindromic repeats
CVD: cardiovascular disease
DAF‐FM: 4‐amino‐5‐methylamino‐2',7'‐difluorofluorescein diacetate
EC: endothelial cell
eNOS: endothelial nitric oxide synthase
HGPS: Hutchinson‐Gilford Progeria Syndrome
HUVEC: human umbilical vein endothelial cell
iPSC: induced pluripotent stem cell
MMP: matrix metalloproteinase
mRNA: messenger RNA
NO: nitric oxide
NOSTRIN: nitric‐oxide synthase trafficking inducer
PARP1: poly (ADP‐ribose) polymerase 1
PECAM1: platelet endothelial cell adhesion molecule 1
RT‐PCR: reverse transcription polymerase chain reaction
SASP: senescence‐associated secretory phenotype
SA‐β‐gal: senescence‐associated beta‐galactosidase
SMA: smooth muscle alpha‐actin
smMHC: smooth muscle myosin heavy chain
SMT: smoothelin
Tie2: tyrosine kinase with immunoglobulin‐like and EGF‐like domains 2
TIMP: tissue inhibitor of metalloproteinases
VCAM1: vascular cell adhesion molecule 1
VEGF: vascular endothelial growth factor
VEGFR‐2: vascular endothelial growth factor receptor 2
VSMC: vascular smooth muscle cell

## 1 INTRODUCTION

The incidence of cardiovascular disease (CVD) increases with age (Rodgers et al., 2019). Age‐associated changes in structure and function of the vasculature elevate the risk of developing CVD (Fleg & Strait, 2012). Therefore, a better understanding of the molecular basis of vascular aging could lead to creating a more effective and targeted therapeutic to treat cardiovascular disease. Accelerated aging models provide a valuable platform for studying vascular aging (Benedicto et al., 2021). Hutchinson‐Gilford progeria syndrome (HGPS) is a rare premature aging disorder caused by a de novo point mutation in exon 11 of the *LMNA* gene (c.1824C>T; p.G608G) (Eriksson et al., 2003). This mutation activates a cryptic splice site, causing a 50 amino acid deletion near the C‐terminus end of the protein product (De Sandre‐Giovannoli et al., 2003; Eriksson et al., 2003). This truncated lamin A variant protein, progerin, remains permanently farnesylated and localizes at the nuclear envelope, interfering with lamin A function in a dominant‐negative manner (Prokocimer et al., 2013). Progerin accumulation at the inner nuclear lamina causes defects in heterochromatin organization, DNA repair, gene expression, and nuclear architecture (Olive et al., 2010; Prokocimer et al., 2013). HGPS patients appear normal at birth; however, within a year or two, they begin to present growth abnormalities such as thin skin, short stature, alopecia, osteoporosis, and progressive atherosclerosis (Olive et al., 2010). Among these clinical features, CVD is the primary cause of death in progeria patients (Hamczyk et al., 2018; Olive et al., 2010).

Endothelial cells (ECs) line the inner surface of blood vessels and play an essential role in maintaining vascular hemostasis and tone (Rajendran et al., 2013). ECs, in direct contact with blood, secrete vasodilators and vasoconstrictors to induce blood vessel relaxation or contraction, respectively (Mudau et al., 2012). EC dysfunction is characterized by impaired vasodilation and deficiency in nitric oxide (NO) formation, contributing to atherosclerosis (Hadi et al., 2005). EC dysfunction is considered an early event in CVD development and is potentially reversible (Medina‐Leyte et al., 2021). Therefore, strategies that can protect or reverse EC dysfunction are at the forefront of CVD preventive efforts (Osmanagic‐Myers et al., 2019).

Studying a mouse model where progerin expression was limited to ECs, Osmanagic‐Myers et al. showed systematic phenotypes of vascular dysfunction, accompanied by cardiac hypertrophy, perivascular and interstitial fibrosis, and premature death (Osmanagic‐Myers et al., 2019). In an in vitro model, tissue‐engineered blood vessels (TEBV) fabricated from healthy iPSC‐derived vascular smooth muscle cells (VSMCs) and HGPS iPSC‐derived ECs showed reduced vasoactivity and increased endothelial cell inflammation (Atchison et al., 2020). Xu et al. compared the cellular dysfunction in human iPSC‐derived ECs and VSMCs from an HGPS patient and an unaffected parent (Xu et al., 2022). They concluded that more extensive cellular dysfunction was observed in HGPS‐ECs than in HGPS‐VSMCs (Xu et al., 2022). These tissue‐specific studies support that progerin expression in ECs could alter active cell–cell communications and exert toxic paracrine effects on other nearby cells.

The endothelium is also involved in forming the blood vessels, which requires significant coordination (McCarron et al., 2017). Maintaining the harmony between the cells undergoing these complex changes depends heavily on the signals exchanged among them (Lilly, 2014). Differentiation and maturation of the vessels are regulated by extrinsic factors, such as cell–cell communication, growth factor gradients, and tissue hypoxia (Li et al., 2018). Therefore, proper communications within ECs are essential for vessel formation and function (Li et al., 2018). Our prior study showed that progerin inhibits endothelial nitric oxide synthase (eNOS) expression and activity in ECs, antagonizing the normal angiogenic function (Gete et al., 2021).

We hypothesized that progerin expression in ECs interferes with their proper communication, leading to vascular dysfunction. In this work, we identify Angiopoietin‐2 (Ang 2) as a dysregulated pro‐angiogenic growth factor whose expression is missing in HGPS ECs and HGPS mice. We showed that Ang2 treatments rescue HGPS ECs tube formation and promote ECs cell migration. The angiogenic effect of Ang2 was abolished by Ang2 inhibitor in control ECs, proving Ang2 is essential for proper tube formation even in control ECs. Ang2 treatments also restore the NO bioavailability in HGPS ECs by activating eNOS. Furthermore, our data suggests that Ang2 therapy alters HGPS ECs paracrine signaling and reverses its detrimental effects. We showed that VSMCs exposed to conditioned media (CM) from HGPS ECs treated with Ang2 addition significantly decreased cell death and cellular senescence. Using an ectopically expressed progerin model and adenine base editor (ABE) corrected ECs, we determined a negative correlation between progerin and Ang2 expression. Elimination of progerin expression by gene editing improves the Ang2 levels. Lastly, our results indicated that Ang2 exerts its beneficial effect in ECs through Tie2 receptor binding, activating an Akt‐mediated pathway. Our investigation demonstrates a novel function for Ang2 or its agonists as a potential therapeutic for HGPS.

## 2 RESULTS

### 2.1 Ang2 expression and secretion are defective in HGPS iPSC‐derived ECs and HGPS animal model aortas

To test our hypothesis and understand the impact of a disease vascular cell type on the neighboring healthy cell type and vice versa in the vasculature, we took advantage of the iPSC‐derived EC model characterized previously by our lab (Gete et al., 2021). In a pilot trial, we examined the interaction between control ECs and HGPS ECs using a conditioned media (CM) experiment. We treated HGPS ECs with EC fresh medium (FM), control EC‐CM, and HGPS EC‐CM for 72 h. HGPS ECs treated with control EC‐CM showed significant downregulation in cell cycle inhibitor genes (p16 and p21) compared to those treated with HGPS EC‐CM. This observation suggested that autocrine signaling molecules from control ECs are potentially responsible for this improvement in HGPS ECs (Figure S1a).

**FIGURE 1:**
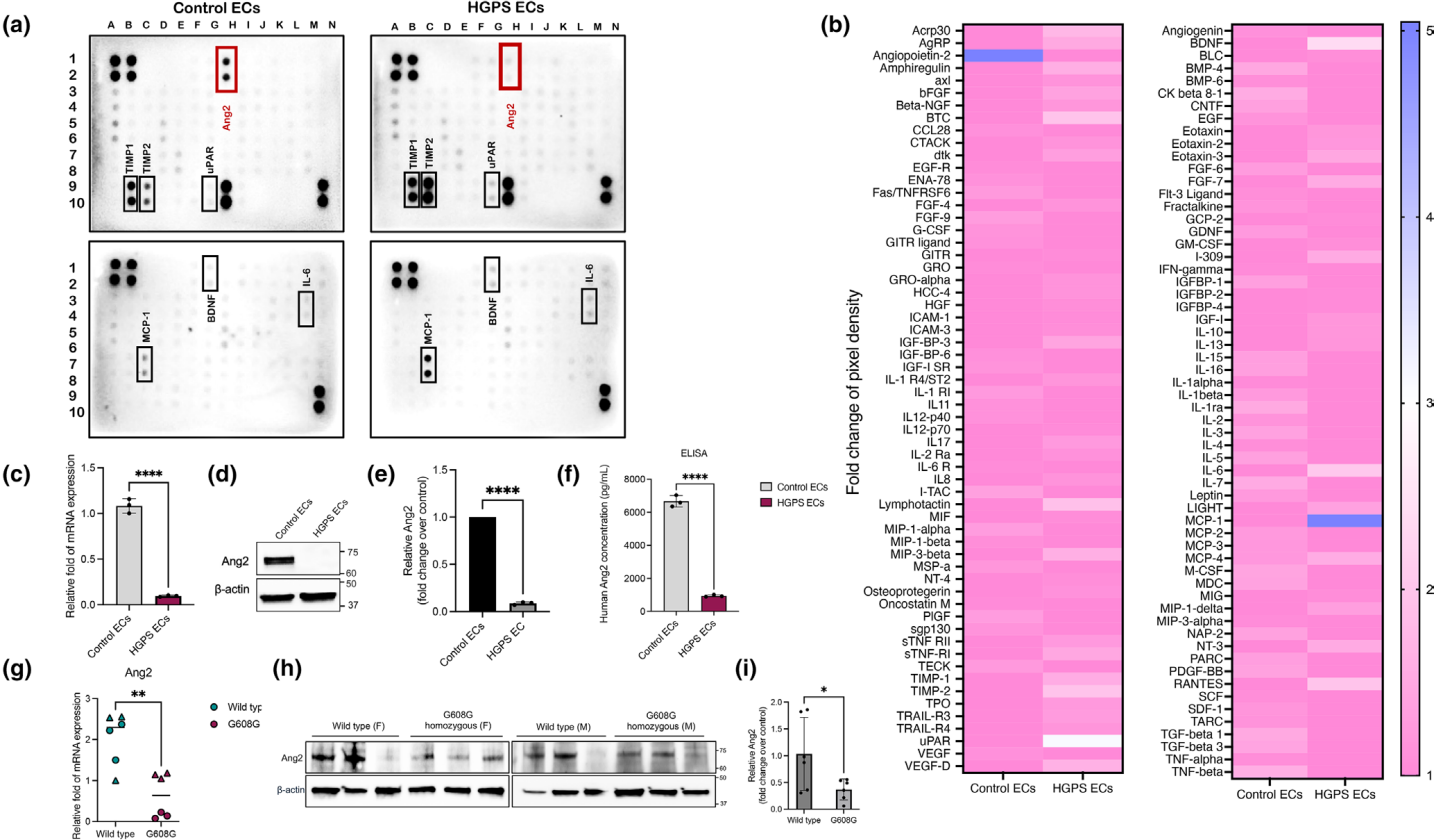
Ang2 expression and secretion are defective in HGPS iPSC‐derived ECs and aortas from an HGPS animal model. (a) A representative array image of the human cytokine antibody array membrane shows the distribution of 120 cytokine capture antibodies immobilized on the membranes. Left panel, control endothelial cells; Right panels, HGPS endothelial cells. The position of each cytokine is shown in Figure S1. (b) Heatmap displaying the mean fold‐change of each cytokine. Data represented the fold change of pixel density in the HGPS ECs group compared with the control ECs group. (c) RT‐QPCR analysis of *Ang2* in control and HGPS ECs. (d) Western blotting analysis with Ang2 antibody on the cell lysates of control and HGPS ECs. (e) Quantification of fold change for the western blot band of the relative Ang2 level normalized to control. (f) Conditioned media Ang2 levels in control and HGPS ECs were detected by enzyme‐linked immunosorbent assay. (g) RT‐QPCR analysis of *Ang2* in wild type and G608G mice aorta (*n* = 6, 4‐month‐old mice per group); triangle symbols correspond to males, and circle symbols correspond to females. (h) Western blotting analysis of the aortic lysates from age‐ and gender‐matched wild‐type and G608G mice with Ang2 antibody (*n* = 6, 4‐month‐old mice per group). (i) Quantification of fold change for the western blot band of the relative Ang2 level normalized to wild‐type. Data are presented as mean ± SEM, **p < 0.01, ***p < 0.001, ****p < 0.0001; *n*, 3 independent experiments.

To identify dysregulated secretory factors caused by progerin accumulation in HGPS ECs, control EC‐CM and HGPS EC‐CM were collected and compared using semi‐quantitative antibody array analysis, which is capable of detecting up to 120 proteins, including chemokines, cytokines, and growth factors (Figure 1a; Figure S1b). This analysis detected a dysregulation in 24 secreted proteins, most of which were overexpressed in HGPS ECs CM compared to control (Figure 1b and Figure S1c,d). Consistent with previous studies, among these differentially secreted molecules, a number of senescence‐associated secretory phenotype (SASP) factors, including tissue inhibitor metalloproteinases 1 and 2 (TIMP1 and TIMP2), IL6, and MCP1, showed higher levels in HGPS ECs relative to healthy controls (Figure 1b) (Csoka et al., 2004; Gete et al., 2021; Lee et al., 2018; Yousefzadeh et al., 2018).

Interestingly, we identified a proangiogenic growth factor, Angiopoietin‐2 (Ang2), to be essentially missing in the secretome of HGPS ECs (Figure 1a,b; Figure S1c,d). Ang2, a growth factor belonging to the angiopoietin/Tie2 pathway, acts as a context‐dependent agonist/antagonist for the Tie2 receptor (Akwii et al., 2019). This molecule is an important mediator of angiogenesis that facilitates vascular endothelial growth factor (VEGF)‐driven angiogenesis (Evans et al., 2017). ECs are the primary source and transcriptional regulators of Ang2 production (Hegen et al., 2004). Ang2 acts in an autocrine manner and function in vascular homeostasis and responsiveness (Hegen et al., 2004).

The above findings led us to consider that progerin accumulation could induce a change in Ang2 levels, contributing to angiogenic defects observed in progeria. To test this possibility, we examined *ANG2* gene expression in control and HGPS ECs. We found a 13‐fold reduction in the transcript level relative to the control (Figure 1c). This reduced transcript abundance in HGPS ECs was followed by a significant decrease in protein abundance relative to healthy controls (Figure 1d,e; Figure S1e,f). The secretion of Ang2 in CM measured using ELISA showed a similar result to the antibody array with a sixfold reduction in HGPS ECs compared to that in control ECs (Figure 1f). We next examined Ang2 expression in the aortic wall of an HGPS mouse model. To directly investigate the Ang2 expression in progeria mice, we carried out Ang2 immunofluorescence analysis in the aorta of 6‐month‐old wild type and G608G homozygous HGPS mouse model. The G608G progeria mouse model, expresses human lamin A, lamin C, and progerin together with endogenous mouse lamin A and C (Cabral et al., 2021; Varga et al., 2006). Both heterozygous and homozygous G608G mice develop a marked vascular phenotype in the aorta and carotid arteries (Cabral et al., 2021; Varga et al., 2006). These include progressive loss of VSMCs, thickening of the adventitia and medial layer, collagen deposition, elastic fiber breakage and vessel calcification in older animals (Cabral et al., 2021; Varga et al., 2006). Notably, we observed that in this HGPS homozygous mouse model, aortic Ang2 expression is significantly lower than the age matched wild types (Figure S1g,h). Ang2 expression was not limited only to ECs, and the signal was distributed throughout the aorta suggesting its release from ECs. In addition, the downregulation of Ang2 mRNA and protein was confirmed in aortic tissue using RT‐QPCR analysis and western blotting analysis, respectively (Figure 1g–i). These observations indicated that Ang2 downregulation is a shared phenotype between the progeria mouse model and the human iPSC‐derived cell model.

### 2.2 Exogenous Ang2 rescues HGPS ECs tube formation defect

**FIGURE 2:**
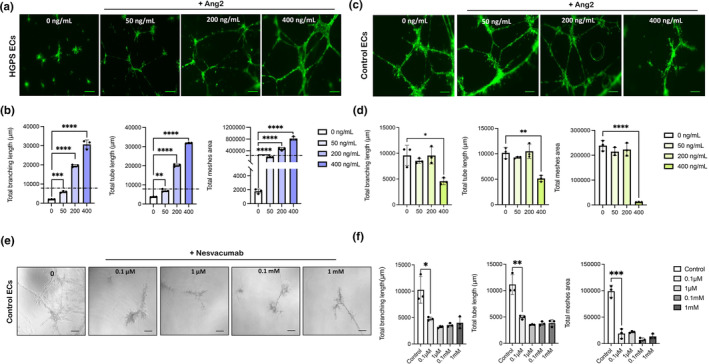
Ang2 treatment rescues capillary‐like microvascular network formation defects in HGPS ECs. Matrigel‐based tube formation assay to assess the angiogenic activity of HGPS and control ECs 18 h after treatment with (0, 50, 200, 400 ng/mL) Ang2 (scale bars = 200 μm). (a) Representative images of tube formation in HGPS ECs after treatment with Ang2. (b) Quantification of total branching length, total tube length, and total meshes areas for *n* = 10 fields of view. The dashed line indicates the control ECs' total branching, tube length, and mesh area. (c) Representative images of tube formation in control ECs after treatment with Ang2. (d) Quantification of total branching length, total tube length and total meshes area for *n* = 10 fields of view. (e) Representative images of tube formation in control ECs after treatment with (0.1 μM, 1 μM, 0.1 mM, 1 mM) Nesvacumab for 18 h (scale bars = 200 μm). (f) Quantification of total branching length, total tube length and total meshes area for *n* = 10 fields of view. Data are presented as mean ± SEM, *p < 0.05; **p < 0.01, ***p < 0.001, ****p < 0.0001; *n*, 3 independent experiments.

Previously, we revealed that HGPS patient‐derived ECs exhibit functional defects in forming capillary‐like microvascular structures (Gete et al., 2021). Tube formation analysis comparing HGPS vs. control ECs after 18 h showed that HGPS ECs form shorter disintegrated tube‐like structures than controls (Figure S2a). HGPS EC tubes exhibited a significant reduction in total tube length, branching length, and total meshing area (Figure S2b). To investigate the potential role of Ang2 on endothelial cell function, we examined the effect of various concentrations of Ang2 on HGPS ECs vasculogenesis. The tube formation assay revealed a significant increase in the microvascular network formation in HGPS ECs in an Ang 2 dose‐dependent manner (Figure 2a). HGPS ECs exposed to 400 ng/mL Ang2 showed the thickest‐cell covered areas, tubes, and branching points compared to cells exposed to a lower dose (Figure 2b). However, even 50 ng/mL of Ang2 rescued tube formation defects observed in HGPS ECs (Figure 2b). Treatment of control ECs with Ang2 concentrations ranging from 50 to 200 ng/mL did not result in any beneficial or harmful effects (Figure 2c). However, higher concentrations of Ang2(400 ng/mL) caused a notable disorganization in the tube‐like structure and a complete abrogation of the network formation in control ECs (Figure 2c,d). This data suggests that high concentrations of Ang2 disrupt control EC function.

To investigate whether functional Ang2 is essential in control EC sprouting and micro vessel formation, we inhibited Ang2 in control ECs. We utilized Nesvacumab, an anti‐Ang2 monoclonal antibody, to block endogenous Ang2 action to examine the impact of Ang2 neutralization in normal vasculogenesis. Treatment with 0.1 μM of Nesvacumab caused partial abrogation of the network and branches between normal ECs (Figure 2e). Nesvacumab remarkably inhibited the formation of tube‐like structures in normal ECs and was not dependent on the dose over the tested range (Figure 2f). Taken together, this data indicates that Ang2 presence and activity is essential for proper tube formation in control and HGPS endothelial cells.

### 2.3 Exogenous Ang2 improves HGPS EC migration and activates genes associated with neovascularization and survival

**FIGURE 3:**
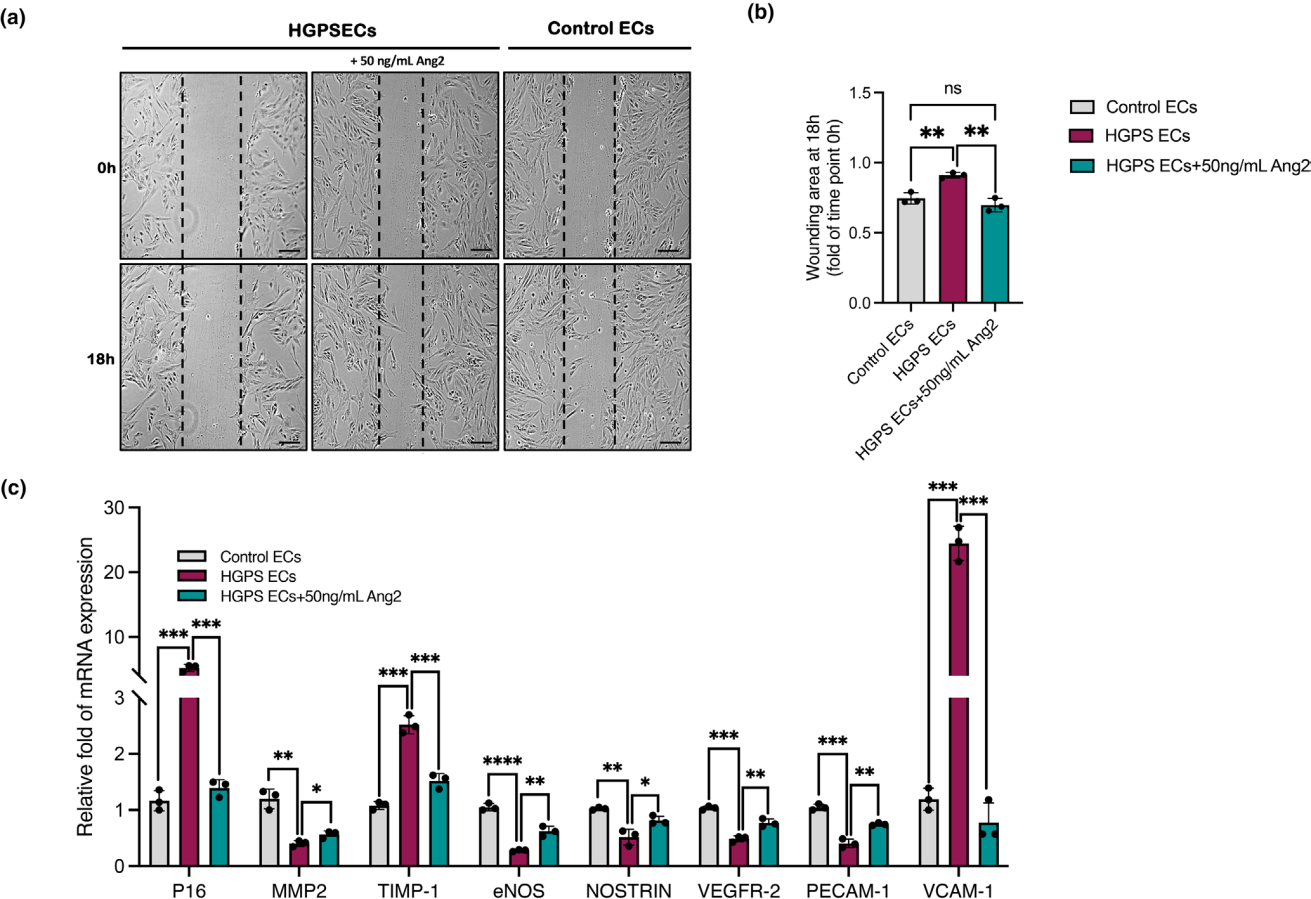
Ang2 improves HGPS ECs migration and activates genes associated with neovascularization. (a) Representative images from in vitro scratch wound healing assays demonstrating that HGPS ECs cell migration into the cell‐free region (outlined) is significantly accelerated in the presence of 50 ng/mL Ang2 when compared to controls (scale bars = 200 μm). (b) Summary bar graph illustrating the ratio of the wounding area at 18 h to the initial area at 0 h. Results represent the mean of 6 measurements of each wounded area, obtained in 3 independent experiments (*n* = 18). (c) RT‐QPCR analysis of P16, MMP2, TIMP‐1, eNOS, NOSTRIN, VEGFR‐2, PECAM‐1, and VCAM‐1 in control and HGPS ECs Ang2 treated group. Data are presented as mean ± SEM, *p < 0.05; **p < 0.01, ***p < 0.001; *n* = 3 independent experiments.

EC migration is essential to vasculogenesis and is tightly regulated by mechanotactic stimuli and chemotactic growth factors (Lamalice et al., 2007). This process requires extracellular matrix degradation to enable the progression of migrating ECs (Deroanne et al., 1997). In a recent study, Jiang et al. revealed that progerin‐expressing ECs significantly reduced the wound healing rate under high shear stress (Jiang & Ji, 2022). This raised the question of whether Ang2 treatment can improve the migration rate of HGPS ECs. To examine the influence of Ang2 on HGPS EC migration, an artificial wound in an HGPS ECs monolayer was induced by scratching the monolayer using a pipet tip. Cells were then treated with recombinant human Ang2, and the wounding area was measured. We used 50 ng/mL Ang2 as this was the lowest concentration that significantly rescued tube formation defects in HGPS ECs. Control ECs wounding area was measured as a positive control. After generating the wound, phase contrast images at different time points were taken, and wound size vs. time was plotted (Figure S3a,b). The slope of the plot showed a significant increase in HGPS ECs migration upon treatment with Ang2 compared to the untreated group (Figure S3b). The wounding area of HGPS ECs 18 h after Ang2 treatment decreased significantly and was comparable to that of control ECs (Figure 3a,b). Given this, we concluded that Ang2 treatment promotes HGPS EC migration.

Next, we investigated the effect of Ang2 treatment on the expression of genes altered by the progerin expression in HGPS ECs utilizing a quantitative RT‐PCR screen. In our previous study, we demonstrated that the expressions of MMP‐2, MMP‐9, PECAM1, and endothelial nitric oxide synthase (eNOS) were down‐regulated in HGPS ECs, while TIMP1 and TIMP2 expression were upregulated (Gete et al., 2021). Quantitative RT‐PCR analysis showed a partial rescue effect in the mRNA level of TIMP‐1, MMP2, eNOS, PECAM1 in the HGPS ECs Ang2 treatment group with no changes observed in MMP9 and TIMP2 levels (Figure 3c; Figure S3c). Upregulation of p16, a well‐known senescent marker, was previously shown in HGPS ECs (Xu et al., 2022). RT‐PCR analysis showed a significant reduction in the p16 mRNA levels after treatment with Ang2 (Figure 3c; Figure S3c). Additionally, we observed a significant upregulation in the mRNA levels of Nitric‐Oxide Synthase trafficking inducer (NOSTRIN) and Vascular endothelial growth factor receptor 2 (VEGFR‐2) (Figure 3c; Figure S3c). Previous studies have shown that vascular cell adhesion molecule 1 (VCAM1), a vascular inflammation marker, expression is elevated in progeria mice and tissue‐engineered blood vessels (viTEBVs) (Atchison et al., 2020; Mojiri et al., 2021). Our RT‐PCR data showed that treatment with Ang2 also reversed the elevated levels of VCAM‐1(Figure 3c; Figure S3c). Importantly, no significant dose‐dependent effect was observed with increasing Ang2 concentrations (Figure S3c). This analysis supports the idea that Ang2 rescues HGPS EC vasculogenesis and migration at least partially through activating signaling molecules and improving survival.

### 2.4 Exogenous Ang2 stimulates NO production and release

**FIGURE 4:**
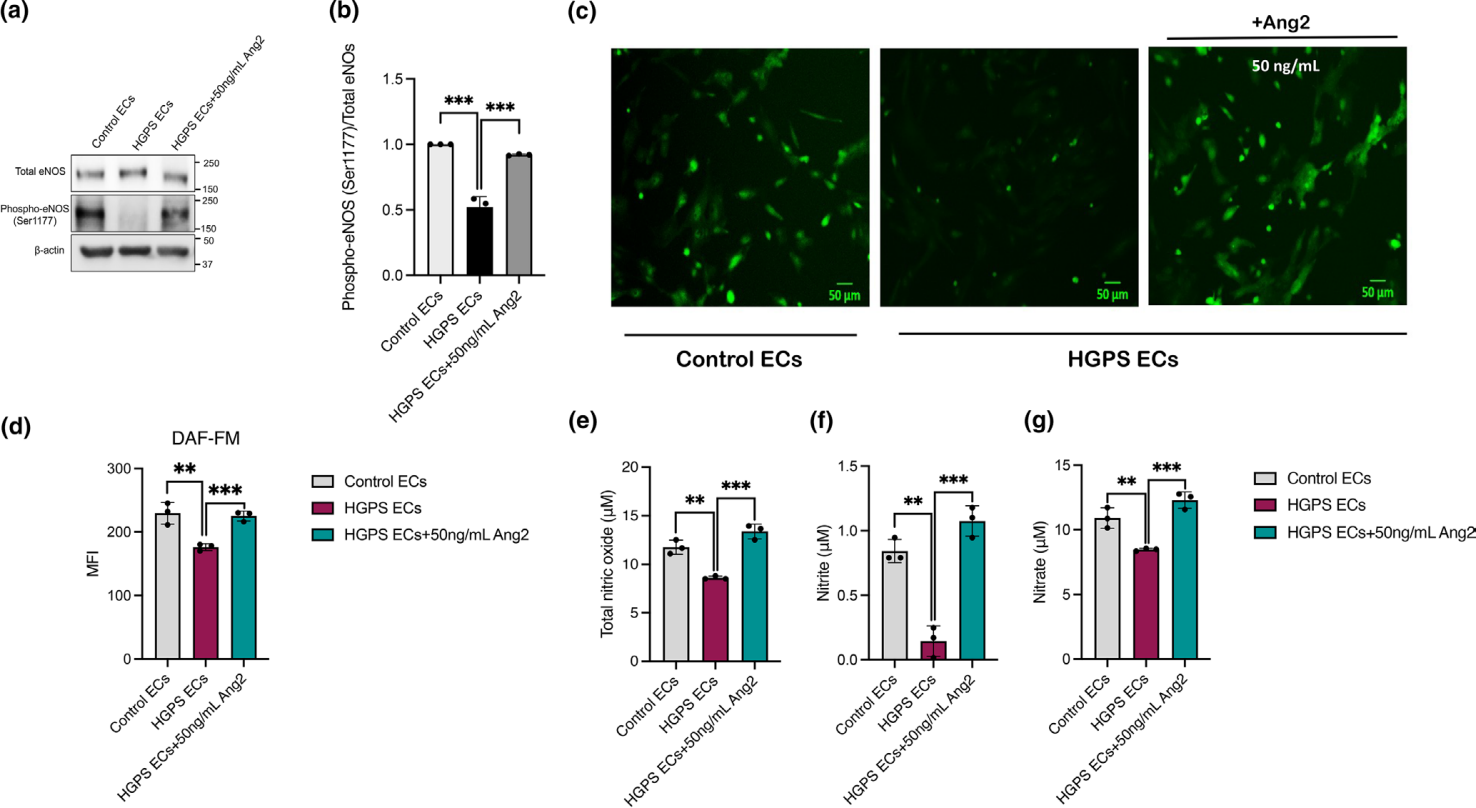
Ang2 stimulates NO production and release in HGPS ECs. (a) Western blotting analysis of the total eNOS and phospho‐eNOS (SER1177) in lysates of control ECs, HGPS ECs, and HGPS ECs treated with 50 ng/mL Ang2. (b) Quantification of fold‐change for relative phosphorylated Ser1177 levels normalized to corresponding total eNOS on immunoblots. (c) Fluorescence images of NO, measured by DAF‐FM staining of control, HGPS ECs, and HGPS ECs treated with 50 ng/mL Ang2 (scale bars = 50 μm). (d) Quantification of the DAF‐FM mean of fluorescence intensity (MFI) for intracellular NO level using FACS. (e–g) Quantification of the extracellular total nitric oxide, nitrite and nitrate in the conditioned media collected from control, HGPS ECs and HGPS ECs treated with 50 ng/mL Ang2. Data are presented as mean ± SEM, *p < 0.05; **p < 0.01, ***p < 0.001, ****p < 0.0001; *n*, 3 independent experiments.

Reduced eNOS activity and expression is linked to impaired neovascularization in HGPS ECs, leading to EC dysfunction (Gete et al., 2021; Xu et al., 2022). Using 4‐amino‐5‐methylamino‐2′,7′‐difluorofluorescein diacetate (DAF‐FM) staining, we showed the intracellular HGPS NO is significantly reduced when compared to controls (Figure S4a). Our quantitative RT‐PCR analysis observed a partial eNOS rescue in Ang2‐treated HGPS ECs (Figure 3c). Given this datum, we hypothesized that Ang2 treatment may restore NO bioavailability in HGPS ECs. To test this hypothesis and check the eNOS function, we measured the abundance of phosphorylated serine 1177 eNOS (Phospho‐eNOS (Ser1177)), a marker of activated eNOS (Chen et al., 2008). In the Ang2‐treated group, the expression of phospho‐eNOS (Ser1177) was significantly higher than in the HGPS ECs control (Figure 4a,b). To further validate our results and quantify intracellular NO levels, we utilized DAF‐FM staining followed by FACS analysis (Figure 4c). Quantification of mean fluorescent intensity (MFI) showed a significant increase in intracellular NO in HGPS ECs after treatment with Ang2 (Figure 4d), which were now comparable to the ECs control. We also measured CM nitrate, nitrite, and total NO concentrations using a colorimetric assay (Arbor Assays). Our results indicated a rescue effect in the total NO, nitrate, and nitrite levels in HGPS ECs CM after treatment with Ang2 (Figure 4e–g). Again, this rescue effect was not dependent on the concentrations of Ang2 over the range tested (Figure S4). Together, this data indicates that exogenous Ang2 stimulates NO production and release when endothelial cell function is compromised.

### 2.5 Exogenous Ang2 improves HGPS ECs paracrine secretome profile

Postmortem studies on HGPS patients and in vivo studies in HGPS transgenic mouse models identified severe VSMC loss in the medial layer of large arteries (Lopez‐Candales et al., 1997; Olive et al., 2010; Varga et al., 2006; Villa‐Bellosta et al., 2013). Mojiri et al. have previously shown that the medial atrophy seen in progeria is in part due to the paracrine effects of ECs SASP in HGPS (Mojiri et al., 2021). Since Ang2 therapy induces NO production and reduces senescence in HGPS ECs, we examined whether this could confer beneficial effects on HGPS VSMCs.

**FIGURE 5:**
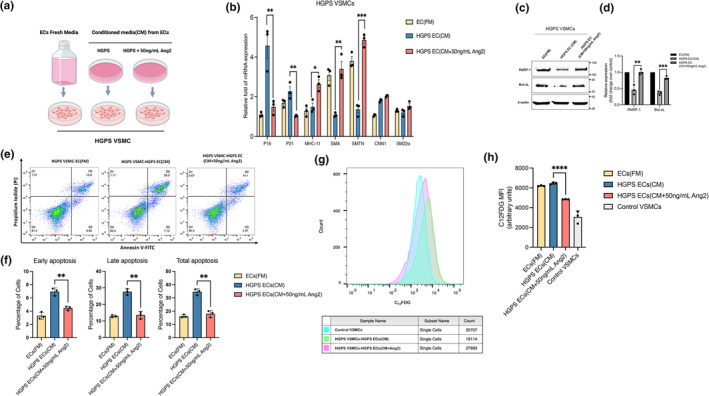
Ang2 improves HGPS ECs paracrine signaling. (a) Conditioned media experimental design. (b) RT‐QPCR analysis of p16, p21, MHC‐11, PARP‐1, SMA, SMTN, Calponin, and SM22α in HGPS SMCs treated with EC fresh media (FM), HGPS ECs conditioned media (CM) and HGPS ECs treated with Ang2 conditioned media (CM + 50 ng/mL). (c) Western blotting analysis of the PARP‐1 and Bcl‐xL from lysates of HGPS SMCs treated with EC fresh media (FM), HGPS ECs conditioned media (CM) and HGPS ECs treated with Ang2 conditioned media (CM + 50 ng/mL). (d) Quantification of fold‐change on Western analysis for relative PARP‐1 and Bcl‐XL levels normalized to HGPS SMCs treated with EC fresh media (FM). (e) PI‐annexin V flow cytometry analysis of HGPS SMCs treated with EC fresh media (FM), HGPS ECs conditioned media (CM) and HGPS ECs treated with Ang2 conditioned media (CM + 50 ng/mL). The gates were set according to the positive and negative controls as suggested by the manufacturer. The cells in the lower right quadrant were scored as the early apoptotic cells, and those in the upper right quadrant were scored as the late apoptotic cells. (f) The percentage of early, late and total apoptotic cells. Approximately 20,000 events were recorded and analyzed per individual sample using Flowjo software. (g) The representative histogram shows C_12_FDG staining and gating on the normal SMCs, HGPS SMC‐HGPS EC (CM), and HGPS SMC‐HGPS EC (CM+ Ang2). (h) Quantification of the C_12_FDG mean fluorescence intensity (MFI). Data are presented as mean ± SEM, *p < 0.05; **p < 0.01, ***p < 0.001, ****p < 0.0001; *n*, 3 independent experiments.

To answer this question, we performed a conditioned media experiment and treated HGPS VSMCs with fresh EC growth medium (FM), HGPS EC‐conditioned medium (CM), and the conditioned media collected from HGPS ECs treated with 50 ng/mL Ang2 (CM + Ang2) for 72 h (Figure 5a). Using a quantitative RT‐PCR screen, we found that Ang2treated HGPS EC‐CM is able to induce a decrease in the expression levels p16 and p21 (cell cycle inhibitors) in HGPS VSMCs compared with those treated with HGPS EC‐CM (Figure 5b). Additionally, we observed an increase in the expression of VSMC specific markers such as SMC–α‐actin (SMA), smoothelin (SMT) and smooth muscle myosin heavy chain (smMHC) (Figure 5b). Notably, there were no significant changes observed in the transcript levels of calponin (CNN1) or SM22‐α, suggesting those markers may need prolonged exposure to show significant changes. Previously, our group demonstrated a critical role of PARP1 in mediating VSMC loss in progeria (Zhang et al., 2014). We showed that progerin accumulation downregulates PARP1 protein levels, which consequently triggers activation of the error‐prone nonhomologous end‐joining response and, eventually, death in HGPS VSMCs. To further investigate the beneficial effects of Ang2 treated ECs on VSMCs, we measured PARP1 and Bcl‐xL protein levels in these cells. Both PARP1 and Bcl‐xL protein levels are rescued after treating HGPS VSMCs with the CM collected from HGPS EC treated with Ang2 (Figure 5c,d). We next examined whether the PARP1 rescue after Ang2 therapy was relevant to the reduction of cell death in HGP VSMCs. To analyze cell death in these HGPS VSMCs, we performed propidium iodide (PI)‐annexin V assay in these treatment groups. FACS analysis of HGPS VSMCs treated with HGPS EC‐CM displayed a two‐fold increase in early, late, and total apoptosis compared to those in control (Figure 5e,f). By contrast, CM from HGPS ECs treated with Ang2 did not elevate apoptosis. In fact, the fraction of apoptotic cells dropped dramatically after treatment with CM collected from the Ang2‐treated group (Figure 5e,f). In addition, to quantify the number of senescent cells in different treatment groups, we used a common flow cytometric assay C_12_FDG that measures senescence‐associated beta‐galactosidase (SA‐β‐gal) activity. Based on the mean fluorescence intensity of C_12_FDG, we identified an elevated SA‐β‐gal activity in the HGPS EC‐CM treated group when compared to those from control VSMC, whereas HGPS VSMCs exposed to CM from the Ang2‐treated HGPS ECs manifested a significant decrease SA‐β‐gal activity (Figure 5g,h). Together, these data suggest that the secretome of Ang2‐treated ECs can benefit the VSMCs' survival and maturity.

### 2.6 Negative correlation between progerin and Ang2 expression

**FIGURE 6:**
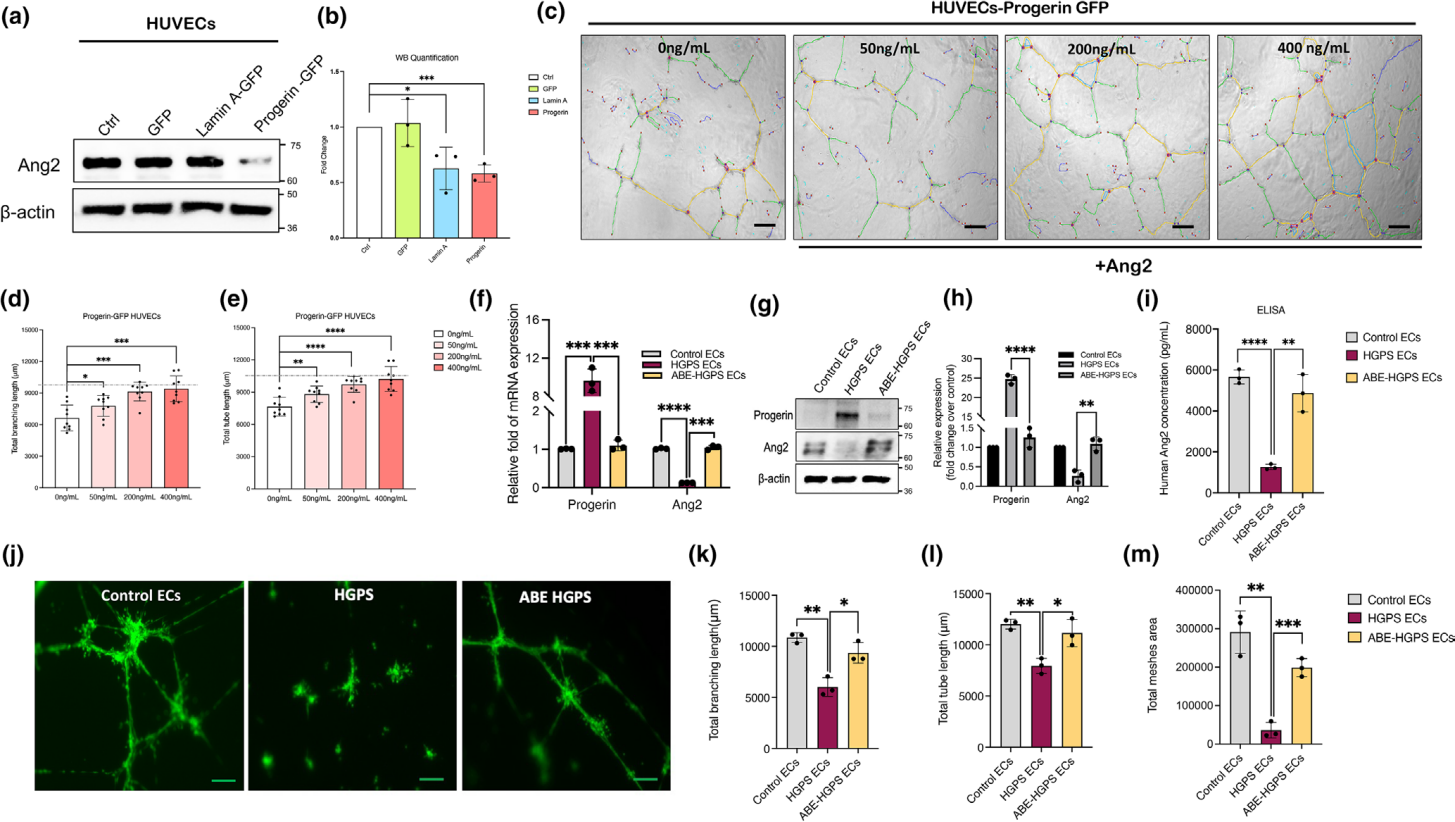
Inverse correlation between progerin and Ang2. (a) Western blotting analysis with indicated antibodies on the lysates of HUVECs transduced with GFP‐control, GFP‐lamin A, or GFP‐progerin vectors. (b) Quantification of fold‐change for western blot band densitometry of the relative Ang2 level normalized to control. (c) Representative images of tube formation in progerin expressed HUVEC after treatment with (0, 50, 200, 400 ng/mL) Ang2 (scale bars = 200 μm). (d) Quantification of total branching length for *n* = 10 fields of view. (e) Quantification of total tube length area for *n* = 10 fields of view. Dashed line indicates the control total branching length and tube length. (f) RT‐QPCR analysis of progerin and Ang2 in control, HGPS, and ABE‐corrected HGPS ECs. (g) Western blotting analysis with indicated antibodies on the lysates of HGPS and ABE‐corrected HGPS ECs. (h) Quantification of fold‐change for western blot band densitometry of HGPS and ABE‐HGPS progerin and Ang2 levels normalized to healthy control. (i) Conditioned media Ang2 levels in normal and HGPS ECs and ABE corrected HGPS ECs were detected by enzyme‐linked immunosorbent assay. (j) Representative images of tube formation in normal, HGPS and ABE corrected HGPS ECs (scale bars = 200 μm). (k) Quantification of total branching length, (l) total tube length, and (m) total meshes area for *n* = 10 fields of view. Data are presented as mean ± SEM, *p < 0.05; **p < 0.01, ***p < 0.001, ****p < 0.0001; *n*, 3 independent experiments.

To determine whether the Ang2 expression defects in ECs were directly induced by progerin, we ectopically expressed progerin in human umbilical vein endothelial cells (HUVECs). We transduced HUVECs using lentiviruses expressing GFP‐progerin, GFP‐lamin A fusion proteins, and GFP only control (Figure S5a,b). Progerin‐expressing HUVECs formed a shorter and less stable microvascular network than the lamin A‐expressing and GFP‐expressing HUVECs (Figure S5c,d). Interestingly, lamin A‐expressing HUVECs also showed a moderate tube formation defect (shorter tube length and branching point) compared to GFP‐expressing HUVECs (Figure S5c,d). Progerin‐expressing HUVECs significantly reduced Ang2 protein levels compared with GFP‐expressing controls (Figure 6a). To confirm whether the toxic effects of progerin are reversible by delivering exogenous Ang2, we treated the progerin‐expressing HUVECs with different doses of Ang2 for 18 h and measured their tube forming capacity. Similar to those seen in iPSC‐derived HGPS ECs, the rescue effects were observed in progerin‐expressing HUVECs treated with Ang2 where total tube and branching length were significantly improved compared to the untreated group (Figure 6c–e).

To answer whether progerin clearance restores Ang2 levels, we used an adenine base editor (ABE7.10max‐VRQR) corrected HGPS iPSCs model. Quantitative RT‐PCR and western blotting analysis showed a complete rescue in Ang2 mRNA levels and a significant increase in Ang2 protein levels upon progerin clearance (Figure 6f–h). Ang2 secretion level in ABE‐corrected HGPS EC conditioned media was measured using ELISA and revealed a significant increase in Ang2 concentration (Figure 6i). Tube formation analysis of ABE‐corrected HGPS ECs also showed a full rescue in the tube forming capacity, suggesting progerin induces angiogenic defects by down‐regulating Ang2 levels (Figure 6j–m). Together, this data suggests a negative correlation between progerin and Ang2 expression.

### 2.7 The protective role of Ang2 in ECs is Tie2 dependent

**FIGURE 7:**
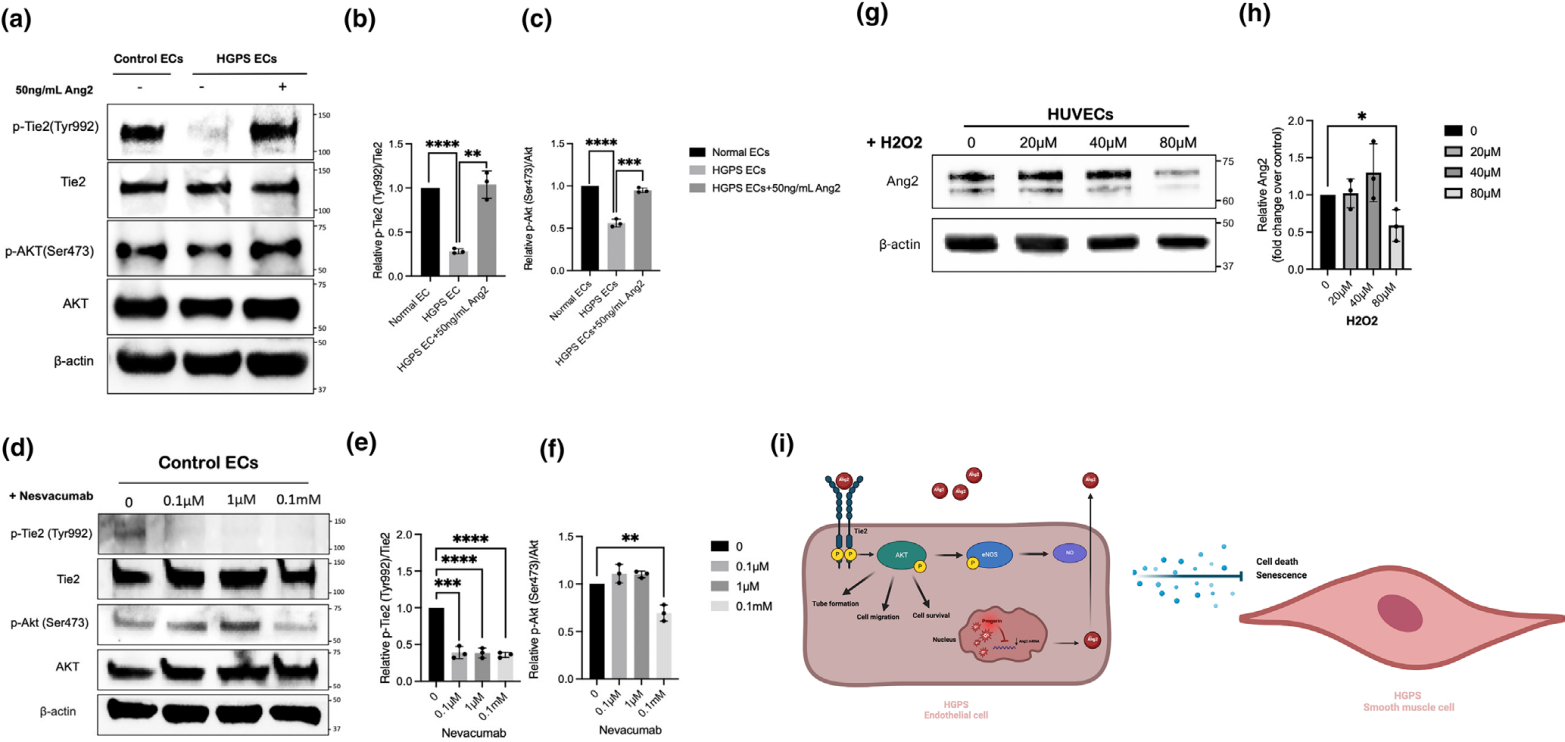
Ang2 protective action is Tie2 dependent. (a) Western blotting analysis with indicated antibodies on the lysates of control ECs, HGPS ECs, and HGPS ECs treated with 50 ng/mL Ang2 for 72 h. (b) Quantification of fold‐change for western blot band densitometry of relative phosphorylated Tyr992 level normalized to corresponding total Tie2. (c) Quantification of fold‐change for western blot band densitometry of relative phosphorylated Ser473 AKT level normalized to corresponding total AKT. (d) Western blotting analysis with indicated antibodies on the lysates of control ECs treated with (0.1 μM, 1 μM, 0.1 mM, 1 mM) Nesvacumab for 24 h. (e) Quantification of fold‐change for western blot band densitometry of relative phosphorylated Tyr992 level normalized to corresponding total Tie2. (f) Quantification of fold‐change for western blot band densitometry of relative phosphorylated Ser473 AKT level normalized to corresponding total AKT. (g) Western blotting analysis with indicated antibodies on the lysates of HUVECs treated with (0 μM, 20 μM, 40 μM, 80 μM) H_2_O_2_ for 24 h. (h) Quantification of fold change for western blot band densitometry of Ang2 level normalized to control. Data are presented as mean ± SEM, *p < 0.05; **p < 0.01, ***p < 0.001, ****p < 0.0001; *n*, 3 independent experiments. (i) Proposed working model. Progerin accumulation induces stress, leading to attenuation of Ang2 expression and secretion and its downstream effector. Ang2‐induced activation of Tie2 receptor activates AKT signaling pathways, which improves HGPS ECs NO production, angiogenesis, survival, migration, and secretome.

To determine possible pathways for beneficial effects mediated by Ang2, we examined Tie2 tyrosine phosphorylation in response to increasing concentrations of Ang2. Tie2 is an EC–specific tyrosine kinase receptor that has been identified as the main receptor in the ANG/TIE signaling pathway for all angiopoietins (Davis et al., 1996; Maisonpierre et al., 1997). Upon treating HGPS CEs with different dosages of Ang 2 for 24 h, we found that Tie‐2 phosphorylation peaked at the 50 ng/mL treatment group, indicating maximal levels of Tie2 activation in that dose (Figure S6a,b). To determine whether the Tie2 downstream effector Akt is involved in the Ang2 beneficial effect, Akt phosphorylation at Ser473 (active Akt) was examined in these samples. No changes were observed in the degree of phosphorylation at 24 h (Figure S6a,c). It is possible that the phosphorylation of Akt, which is downstream of Tie 2, may need more time to be detected. Therefore, we treated HGPS ECs with 50 ng/mL Ang2 for 72 h. Extended treatment of HGPS ECs with Ang2 significantly increased Tie2 phosphorylation and Akt activation (Figure 7a–c).

The downgrading of Tie2 signaling is thought to lead to vessel destabilization (Bogdanovic et al., 2006). As shown in Figure 2e,f, Nesvacumab anti‐Ang2 monoclonal antibody disrupted vasculogenesis in the control ECs. To investigate the impact of Ang2 neutralization on the Tie2 activation, control ECs were treated with an increasing dose of Nesvacumab for 24 h. Nesvacumab inhibited Tie2 autophosphorylation; consistent with our previous observations, this inhibition was not dependent on the dose (Figure 7d–f). The highest dose of Nesvacumab (0.1 mM) significantly decreased AKT S473 phosphorylation (Figure 7d–f). These results indicate that Ang2 protective effects are in part due to activating Tie2 and bolstering the pro‐survival AKT pathway.

Previous studies have shown that progerin expression in ECs induces oxidative stress, as well as cellular senescence and DNA damage which further contributes to ECs dysfunction (Bidault et al., 2020; Burtenshaw et al., 2019; Hashimoto et al., 2017). To further investigate whether oxidative stress‐induced cellular senescence causes Ang2 reduction, we treated HUVECs with increasing concentrations of Hydrogen peroxide (H_2_O_2_) (20, 40, 80 μM) for 24 h. Western blot analysis of the HUVECS showed a significant reduction in the expression levels of Ang2 at the 80 μM H_2_O_2_ treated group (Figure 7g,h), suggesting excessive oxidative stress could induce translational attenuation of Ang2.

## 3 DISCUSSION

ECs play a vital role in atherosclerosis prevention. However, very little is known about the molecular mechanisms that confer atheroprotection (Rakhit & Marber, 2001). Most previous studies on HGPS EC signaling focus on the deleterious impact of senescent cells and their senescence‐associated secretory phenotypes (SASP), which deteriorates the microenvironment and impairs cellular functions in nearby cells (Coppe et al., 2010). Barinda et al. reported that EC senescence impairs systemic metabolic health through adipose tissue dysfunction in EC‐specific progeroid mice (Barinda et al., 2020). Bidault et al. showed that progerin expression in ECs is sufficient to induce an inflammatory response and to increase adhesion molecules expression, which would further recruit macrophages and form atherosclerotic plaques in HGPS patients (Bidault et al., 2020). Using an endothelium‐specific HGPS mouse model, Manakanatas et al. reported that progerin‐expressing ECs initiate a senescence response in non‐EC populations and cause immune cell infiltration around blood vessels (Manakanatas et al., 2022). While these studies support senescence‐induced detrimental effects in ECs, they focus less on the potential protective effect of cells that do not express progerin.

ECs secrete a vast range of substances that play an important role in inducing EC mobilization, proliferation, and migration. Koblan et al. reported CRISPR/Cas9‐mediated ABE that convert A·T to G·C in genomic DNA can efficiently correct the HGPS mutation and rescue the phenotypes (Koblan et al., 2021). Surprisingly, in some tissues, such as the aorta, even modest DNA editing resulted in excessively large benefits at the RNA, protein, and tissue levels (Koblan et al., 2021). ~25% correction in the aorta was enough to almost fully rescue VSMC counts, adventitia thickness, and extension of the life span of a progeria mouse model to nearly the normal range (Koblan et al., 2021). This data suggested that the unedited VSMC cells senesced and that the edited cells had enough replicative potential to take up their space. In addition to this in vivo selection, the correction of mutation in HGPS ECs may improve their cell signaling to the surrounding cells and contribute to the overall health of the tissue. Proper tissue hemostasis is dependent on proper bidirectional communication between cells. Therefore, it is reasonable to expect an exchange of signaling molecules between healthy and neighboring unhealthy cells to occur. Given the location of ECs in the innermost layer of the blood vessel, preferential reception of various stimuli, and their communication with VSMCs, we decided to test this hypothesis in these cells. In this study, we identified Ang2 as a missing factor in the HGPS ECs secretome (Figure 1; Figure S1). Ang2 is a context‐dependent agonist/antagonist ligand for the Tie2 receptor that facilitates vascular endothelial growth factor (VEGF) associated angiogenesis (Holash et al., 1999). High concentrations of Ang2 induce Tie2 phosphorylation and, therefore, prevent EC apoptosis by activating the downstream pro‐survival AKT pathway, which in turn phosphorylates eNOS (Dimmeler et al., 1999; Teichert‐Kuliszewska et al., 2001). A significant downregulation of Ang2 mRNA and protein was observed in HGPS mouse aortic tissue (Figure 1g–i). We noticed that two wild‐type samples had lower expression of Ang2 than the rest of the group, which may reflect the heterogeneous stress levels in wild‐type animals or during sample preparations.

In this study, we demonstrate that progerin‐dependent angiogenesis deficiency in HGPS ECs is reversible by Ang2 treatments that induce the formation of extensive capillary‐like tubes (Figure 2a,b). Notably, this rescue effect was dose‐dependent (Figure 2a,b), and none of the other beneficial effects of Ang2 treatments we observed in this study were dose‐dependent. The potential mechanism for this could be that Ang2 is reported to bind to ECs αvβ3, αvβ5, and α5β1 integrins with lesser affinity, in addition to Tie2. This binding activates angiogenesis in a Tie2‐independent manner (Felcht et al., 2012). By increasing Ang2 concentrations, we may induce angiogenesis through both Tie2 dependent and Tie2 independent pathways and, therefore, observe dose‐dependent angiogenesis. We provided further evidence that the protective role of Ang2 in HGPS ECs depends on Tie2 /Akt signaling pathways (Figure 7a–c). This was confirmed by the Ang2 inhibition in control ECs, which effectively inhibited their angiogenesis through Tie2 /Akt deactivation (Figure 7d–f).

No changes in the degree of angiogenesis were observed in control ECs treated with Ang2 up to a concentration of 200 ng/mL (Figure 2c,d). A higher concentration of Ang2 at 400 ng/mL caused widespread vessel discontinuities and loss of network formation in control ECs, suggesting excessive Ang2 in a normal condition can be deleterious (Figure 2c,d). Similar results were observed after treating control HUVECs using increased doses of Ang2 (data are not shown). That may be because, under certain conditions, excessive amounts of Ang2 can antagonize Angiopoietin‐1(Ang1) function by binding to Tie2 without inducing phosphorylation and disrupting protective Tie2 signaling, destabilizing formed vessels (Yuan et al., 2009). Inhibition of Ang2 caused regression in control ECs tube formation, suggesting Ang2 activity is essential for proper tube formation (Figure 2e,f). Furthermore, we show that Ang2 treatment ameliorates impaired wound healing observed in HGPS ECs (Figure 3a,b).

Additionally, Ang2 treatments rescued the dysregulation of genes previously shown to be affected by progerin expression, including p16, eNOS, PCAM‐1, MMP2, TIMP1 and others (Figure 3c). The loss of eNOS activity is an established phenotype in HGPS ECs, and endothelium‐derived NO seems to play an important role in atherosclerosis prevention (Gao et al., 2016; Gete et al., 2021; Osmanagic‐Myers et al., 2019). Our data demonstrated that treatment of HGPS ECs with Ang2 resulted in an increase in eNOS activity and NO production and release (Figure 3c and Figure 4). Although other growth factors, such as VEGF and Ang1, are also able to induce NO release, these inductions are accompanied by recruitment of inflammatory cells (Ahmed et al., 2009). Ahmed et al. reported that Ang2 inhibits atherosclerotic plaque formation in apoE^−/−^ mice by reducing macrophage accumulation and LDL oxidation (Ahmed et al., 2009). This ability of Ang2 to stimulate NO production and release without recruiting inflammatory cells in vivo would make it a perfect atheroprotective factor for HGPS vasculature. Furthermore, we showed that treatment of HGPS ECs with Ang2 alters the EC secretive to improve HGPS VSMC survival and function (Figure 5). Ang2 treatment causes a systemic improvement in HGPS EC health and therefore changes these cells' secretome signature, thus sending a better signal to HGPS SMCs. This improvement can be explained by the fact that treatment of HGPS ECs with Ang2 reduces the SASPs (p16, MMP2, TIMP1) and alters the composition of their secretome (Figure 3c), with beneficial consequences in the neighboring cells (HGPS SMCs).

To directly test whether progerin expression results in Ang2 downregulation in ECs under the same genetic background, we used HUVECs that ectopically express progerin and ABE‐corrected HGPS ECs (Figure 6). We showed that progerin expression directly causes Ang2 downregulation and therefore induces tube formation deficiency (Figure 6). Moreover, treatment with recombinant Ang2 rescued the tube formation defects observed in progerin expressing HUVECs in a dose‐dependent manner. Interestingly, our data revealed minor tube formation defects in wild‐type lamin A expressed group (Figure S5c–e). However, the magnitude of this defect was less than that seen with progerin. These observations can be explained by the sensitive nature of endothelial cells to lamin A/C imbalance, which in turn can impact regulatory pathways and gene expression (Alcorta‐Sevillano et al., 2020). To promote cell migration, cells require soft nuclei with low lamin A content (Alcorta‐Sevillano et al., 2020). Several studies have demonstrated that overexpression of lamin A increases nuclear stiffness, consequently limiting the migration of ECs similar to those observed in progerin expression (Davidson et al., 2014; Denais & Lammerding, 2014; Friedl et al., 2011). Based on these observations, it is tempting to speculate that lamin A/C imbalance causes EC nuclei to become stiffer and, therefore, less able to migrate and form tubes. Progerin correction, on the other hand, restores the Ang2 mRNA, protein, and secretion level in HGPS ECs and normalizes their tube‐forming capacity (Figure 6e–i). These above experiments indicate that progerin induces Ang2 downregulation in ECs, while the mechanism by which this mechanism occurs in HGPS ECs requires further investigation. Given that environmental stress seems to attenuate Ang2 expression, we speculate that stress‐induced premature senescence caused by progerin may at least partially contribute to Ang2 downregulations (Figure 7g,h).

We propose a model (Figure 7i) in which progerin accumulation increases endothelial cell stress, leading to down‐regulation of Ang2 expression. Since the Ang2/Tie2/AKT pathway is involved in the transcriptional regulation of many genes involved in EC survival, proliferation, migration, chemotaxis, and tube formation, Ang2 attenuation in HGPS causes EC dysfunction (Yuan et al., 2009). In addition, we showed that Ang2 treatment reversed progerin‐induced EC dysfunction by improving HGPS ECs vasculogenesis, migration, survival, NO release, and paracrine secretory profile. To conclude, the remarkable improvements in HGPS ECs after a single treatment with Ang2 collectively suggest that this growth factor may have therapeutic potential to treat vascular disease in HGPS.

## 4 METHODS

### 4.1 Endothelial cell and smooth muscle cell differentiation

Human iPSC line HGPS HGADFN167, HGADFN155 and control HGFDFN168 (father of HGADFN167) with the classic G608G HGPS mutation were obtained from Progeria Research Foundation Cell and Tissue Bank and were used for ECs differentiation. For ABE‐corrected EC differentiation HGPS HGADFN167 iPSCs transfected with lentivirus containing ABE7.10max was used. iPSC lines were differentiated to ECs using a previously established protocol with minor modifications in Refs Atchison et al. (2020) . iPSCs were cultured in mTeSR plus medium (STEMCELL Technologies) and were passaged upon 70% confluency. At passage 28 monolayer iPSCs were cultured in DMEM/F12 (DMEM/F12; Life Technologies, 11,320–033), supplemented with 5 μM CHIR 99021, (Cayman, 13,122), 25 ng/m bone morphogenetic protein‐4 (PeproTech, 120‐05), N2 supplement (Life Technologies, 17,502,048), and B27 supplement (Life Technologies, 12,587,010) for 3 days. At day 3, media was changed to StemPro media (Life Technologies, 10639‐011) supplemented with 5 μM forskolin (Abcam, ab120058), and 100 ng/mL vascular endothelial growth factor (VEGF) (PeproTech, 100–20) for 2 days. On day 7, ECs were cultured on the fibronectin‐coated plate (Corning, 356008) in StemPro media supplemented with 50 ng/mL VEGF and maintained at 37°C and 5% CO_2_ in a humidified incubator. Cells were passaged once they reached 70%–80% confluency.

For SMC differentiation, iPSCs were cultured in mTeSR plus medium (STEMCELL Technologies) and were passaged upon 70% confluency. Monolayer iPSCs at passage 25 were cultured in N2B27 media consist of DMEM/F12 (DMEM/F12; Life Technologies, 11,320–033) and Neurobasal medium (Life Technologies) at a 1:1 ratio, supplemented with 5 μM CHIR 99021, (Cayman, 13,122), 25 ng/m bone morphogenetic protein‐4 (Peprotech, 120–05), 1% N2 supplement (Life Technologies, 17,502,048), and 2% B27 supplement (Life Technologies, 12,587,010) and 0.1% β‐mercaptoethanol (Life Technologies) for 3 days. At day 3, media was replaced with SMC induction media containing N2B27 supplemented with 10 ng/mL PDGF‐BB(Peprotech) and 2 ng/mL Activin A (Peprotech) for 2 days. On day 5, SMCs were removed and cultured on the collagen‐coated plate (Corning, CLS354236) in N2B27 media supplemented with 10 ng/mL PDGF‐BB(Peprotech) and 2 ng/mL Activin A (Peprotech) and maintained at 37°C and 5% CO_2_ in a humidified incubator. Cells were passaged once they reached 70%–80% confluency.

### 4.2 *LMNA* transgenic mouse model

The HGPS transgenic mouse model was developed by retrofitting a human bacterial artificial chromosome (BAC) harboring the *LMNA* gene containing the classic G608G mutation (c.1824C>T) as previously described (Cabral et al., 2021; Varga et al., 2006). Animal care and experiments were performed in accordance with a protocol (NHGRI G03‐5) approved by the NHGRI Animal Care and Use Committee. Mice were housed in individually ventilated cages with a 12‐h light/dark cycle. Standard NIH31 chow and water were provided ad libitum following weaning at 3 weeks of age.

### 4.3 Immunofluorescent staining of aorta

Immunohistostaining was performed following the protocol previously described (Varga et al., 2006) with minor modifications, and by using mouse monoclonal anti‐lamin A/C (Anti‐Lamin A + Lamin C antibody [JOL2], ab40567; 1:75 dilution) antibody or rabbit polyclonal anti‐Ang2 antibody (Angiopoietin‐2(D200); Cell Signaling, 1:100). Briefly, ascending aorta sections were dewaxed and rehydrated, and the antigens were retrieved by heating in EDTA buffer (1 mM, pH 8.0) for 2 min in a pressure cooker. Tissue sections were blocked in TBS buffer containing 10% donkey serum and 1% BSA for 45 min at RT, and then incubated with a Mouse‐on‐Mouse blocking reagent (Vector Laboratories MKB‐2213‐1) for 60 min to reduce endogenous mouse antibody binding. Slides were incubated with the above primary antibodies overnight at 4°C. After washing thoroughly in TBS (5 times), the sections were then incubated with goat anti‐mouse Alexa Fluor 594‐conjugated or donkey anti‐rabbit Alexa Fluor 488‐conjugated secondary antibodies (ThermoFisher Scientific; 1:3000 dilutions). All tissue sections were mounted in DAPI‐containing medium (Vector Laboratories). Fluorescence images were captured by confocal microscope system (Zeiss LMS 880) with 40× water lens and Zeiss AX10 microscope equipped with a SPOT PURSUIT camera with 5x lens. For IF staining the 6‐month time point was chosen as a time that homozygous G608G mice typically show phenotypic decline but are not yet at the end of their median lifespan.

### 4.4 Protein isolation and western blotting from mouse tissues

After sacrifice, tissues from wild type or G608G mice were flash frozen and stored in liquid nitrogen until the time of protein extraction. To extract proteins, 10–30 mg of frozen tissue was first pulverized in temperature resistant tubes (Covaris, tissueTube #520001) on a liquid nitrogen bath. This frozen tissue powder was resuspended in RIPA lysis buffer, moved to a 2‐mL collection tube, and homogenized for 30 s at 25 Hz with a 5‐mm stainless steel bead in a TissueLyser II (Qiagen). Samples were then centrifuged for 5 s on table‐top microcentrifuge and incubated on ice for 45 min while rocking. After incubation, lysates were clarified by centrifugation at 21,000 g for 15 min at 4°C. The supernatant was transferred to a fresh tube and protein quantified using standard BCA assay. Tissue lysates were prepared identically to the cell line isolated samples and separated by SDS‐PAGE (25 μg protein loaded) on 4%–15% Tris‐Glycine gels (Mini‐PROTEAN, BioRad). Tissue western blots were performed using the same method as cell‐line western blots.

### 4.5 RNA isolation from mouse aortas

Mice aortas were collected into Trizol reagent (ThermoFisher), homogenized and immediately flash‐frozen until ready for total RNA isolation. RNA was subsequently digested for 20 min at 37°C with recombinant DNase I (ThermoFisher), then analyzed for integrity and concentration on an Agilent nucleic acid bioanalyzer (Agilent Technologies). Synthesis of cDNA used 1 μg of RNA, which was reverse transcribed using the iScript cDNA Synthesis kit (BioRad) according to the manufacturer's protocol. Quantitative RT‐PCR was performed in triplicates using SYBR Green Supermix (Bio‐Rad) and was detected using the CFX96 Real‐Time PCR Detection System (C1000 Thermal Cycler, Bio‐Rad). The sequences of primers are listed in the appended table.

### 4.6 Conditioned media experiment

To prepare the conditioned medium (CM), passage 3 subconfluent ECs were given StemPro media supplemented with 50 ng/mL VEGF. The conditioned medium was collected after 24 h of incubation. To prepare the control medium, StemPro media supplemented with 50 ng/mL VEGF was put in a culture dish without cells and incubated in the CO_2_ incubator for 24 h in a parallel way. After the first day of plating ECs or SMCs, the growth medium was replaced with a mixture of fresh growth medium and either of the control or CM derived from HGPS EC or HGPS ECs treated with Ang‐2, and incubated for 72 h with changing the medium at 48 h.

### 4.7 Antibody arrays

Detection of multiple cytokines present in normal and HGPS endothelial cells conditioned media was done using array‐based technology. For this purpose, RayBio C‐Series Human Cytokine Antibody Array 1000 Kit (RayBio AAH‐CYT‐10000‐2) was used. This kit combines two antibody array membranes to detect expression of 120 cytokines. The experiment was performed according to the manufacturer's instructions. Briefly, after a 30‐min incubation with Blocking Buffer, 1 mL of control and HGPS ECs conditioned media were added to the arrays and incubated overnight at 4°C on a plate shaker. Chemiluminescence signals were detected after biotinylated antibody cocktail incubation followed by HRP‐Streptavidin incubation. The intensity of the signal for each antibody spot is proportional to the relative concentration of the antigen in that sample. Data analysis was performed to measure the relative differences in expression levels of each antigen between two groups. Once the raw numerical densitometry data was extracted, the background was subtracted and the data were normalized to the positive control signals.

### 4.8 Western blotting

Whole‐cell lysates for immunoblotting were prepared by lysing the cell in Laemmli sample buffer containing 5% of 2‐mercaptoethanol (Bio‐Rad). Samples were boiled at 95°–100°C for 5 min and loaded onto 4 to 15% SDS–polyacrylamide gel electrophoresis (4561086, Bio‐Rad) and transferred onto 0.45 μm pore‐size nitrocellulose membranes (Bio‐Rad) using the Turboblot (BioRad). Blots were blocked with 5% milk for 1 h at room temperature and were then incubated with anti‐lamin A/C (MAB3211; Millipore, 1:500 dilution), progerin (Cao et al., 2011) with a dilution of 1:500, anti‐eNOS (D9A5L; Cell Signaling, 1:1000), anti‐pSer1177 eNOS (Cell Signaling, 1:1000), anti‐angiopoietin‐2 (D200; Cell Signaling, 1:1000), anti‐Tie2(D9D10; Cell Signaling, 1:1000), anti‐pTyr992 Tie2 (4221; Cell Signaling, 1:1000), anti‐pSer473 AKT (D9E; Cell Signaling, 1:1000), anti‐AKT (9272; Cell Signaling, 1:1000), and β‐actin (1:5000, Sigma‐Aldrich). Secondary antibodies include anti‐mouse (sc‐516,102; Santa Cruz, 1:5000), anti‐rabbit (211–035–109; Jackson Immuno‐Research, 1:5000). Protein signals intensity of the experimental target was normalized to housekeeping protein in each lane using image lab.

### 4.9 Elisa

Ang2 levels were measured using BioLegend's ELISA MAX™ Deluxe Set Human Angiopoietin‐2 (BioLegend, 440,404) as per the manufacturer's protocol. The conditioned media from ECs were collected and diluted by 1:3 with a dilution buffer provided by the manufacturer. A plate reader (TermoScientifc) was used to read the absorbance at 450 nm within 15 min.

### 4.10 RNA isolation, cDNA synthesis, and quantitative RT‐ PCR

Total RNA was extracted from ECs using TRIzol® reagent (Life Technologies, 15596026) and purified using the RNeasy Mini kit (Qiagen) as per the manufacturer's instructions. Using the NanoDrop 2000 Spectrophotometer (Termo Fisher Scientifc) RNA yield was determined and 600 μg of total RNA was converted to cDNA using the iScript Select cDNA Synthesis kit (Bio‐Rad). Quantitative RT‐PCR was performed in triplicates using SYBR Green Supermix (Bio‐Rad) and was detected using the CFX96 Real‐Time PCR Detection System (C1000 Thermal Cycler, Bio‐Rad). ΔCt analysis method normalized to GAPDH was carried out to calculate the gene expression. All primers sequences used in this study are listed in Table 1.

**TABLE 1:** Primer sequences used for RT‐PCR experiment.

Target gene	Primer sequence
GAPDH	Forward: 5′‐GTCTCCTCTGACTTCAACAGCG‐3′ Reverse: 5′‐ACCACCCTGTTGCTGTAGCCAA‐3′
LMNA	Forward: 5′‐GCAACAAGTCCAATGAGGACCA‐3′ Reverse: 5′‐CATGATGCTGCAGTTCTGGGGGCTCTGGAT‐3′
Progerin	Forward: 5′‐GCAACAAGTCCAATGAGGACCA‐3′ Reverse: 5′‐CATGATGCTGCAGTTCTGGGGGCTCTGGAC‐3′
p16 ink4a	Forward: 5′‐GAGCAGCATGGAGCCTTC‐3′ Reverse: 5′‐CGTAACTATTCGGTGCGTTG‐3′
p21 CDKN1A	Forward: 5′‐CGATGGAACTTCGACTTTGTCA‐3′ Reverse: 5′‐GCACAAGGGTACAAGACAGTG‐3′
MMP2	Forward: 5′‐AGCGAGTGGATGCCGCCTTTAA‐3′ Reverse: 5′‐CATTCCAGGCATCTGCGATGAG‐3′
MMP9	Forward: 5′‐GCCACTACTGTGCCTTTGAGTC‐3′ Reverse: 5′‐CCCTCAGAGAATCGCCAGTACT‐3′
TIMP‐1	Forward: 5′‐GGAGAGTGTCTGCGGATACTTC‐3′ Reverse: 5′‐GCAGGTAGTGATGTGCAAGAGTC‐3′
TIMP‐2	Forward: 5′‐ACCCTCTGTGACTTCATCGTGC‐3′ Reverse: 5′‐GGAGATGTAGCACGGGATCATG‐3′
eNOS	Forward: 5′‐GAAGGCGACAATCCTGTATGGC‐3′ Reverse: 5′‐TGTTCGAGGGACACCACGTCAT‐3′
Angiopoietin‐2	Forward: 5′‐CAACAGTGTCCTTCAGAAGCAGC‐3′ Reverse: 5′‐CCAGCTTGATATACATCTGCACAG‐3′
Nostrin	Forward: 5′‐AGACCTCAACCCAGCCATCCTT‐3′ Reverse: 5′‐GAAGAACCTGGATTGCTCTGCC‐3′
PECAM‐1	Forward: 5′‐AAGTGGAGTCCAGCCGCATATC‐3′ Reverse: 5′‐ATGGAGCAGGACAGGTTCAGTC‐3′
VEGF‐R	Forward: 5′‐CCTGCAAGATTCAGGCACCTATG‐3′ Reverse: 5′‐GTTTCGCAGGAGGTATGGTGCT‐3′
VCAM‐1	Forward: 5′‐GATTCTGTGCCCACAGTAAGGC‐3′ Reverse: 5′‐TGGTCACAGAGCCACCTTCTTG‐3′
Tie‐2	Forward: 5′‐GGTCAAGCAACCCAGCCTTTTC‐3′ Reverse: 5′‐CAGGTCATTCCAGCAGAGCCAA‐3′
MHC‐11	Forward: 5′‐GTCCAGGAGATGAGGCAGAAAC‐3′ Reverse: 5′‐GTCTGCGTTCTCTTTCTCCAGC‐3′
SMA	Forward: 5′‐GTCCAGGAGATGAGGCAGAAAC‐3′ Reverse: 5′‐CTATGCCTCTGGACGCACAACT‐3′
PARP1	Forward: 5′‐GACCTGAAGGAGCTACTCATC‐3′ Reverse: 5′‐TTTCTCGGAATTCCTTTGGGG‐3′
Smoothelin (SMTN)	Forward: 5′‐CCGAGTGAACAAAGCACCAGAAG‐3′ Reverse: 5′‐TTCCGCTCTTCAAAGTCCGTGC‐3′
Calponin	Forward: 5′‐CCAACGACCTGTTTGAGAACACC‐3′ Reverse: 5′‐ATTTCCGCTCCTGCTTCTCTGC‐3′
SM22α	Forward: 5′‐TCCAGGTCTGGCTGAAGAATGG‐3′ Reverse: 5′‐CTGCTCCATCTGCTTGAAGACC‐3′
MOUSE Ang2	Forward: 5′‐AACTCGCTCCTTCAGAAGCAGC‐3′ Reverse: 5′‐TTCCGCACAGTCTCTGAAGGTG‐3′
MOUSE GAPDH	Forward: 5′‐CATCACTGCCACCCAGAAGACTG‐3′ Reverse: 5′‐ATGCCAGTGAGCTTCCCGTTCAG‐3′

### 4.11 Tube formation assay

96‐well cell culture plates were coated with 10 mg/mL (Corning Matrigel Basement Membrane Matrix Growth Factor Reduced, Phenol Red Free, 356,231). 300 μL of the cell suspension 20,000 cells were seeded into each well, and the plates were incubated for 18 h at 37°C, 5% CO_2_ atmosphere. Cells were labeled with Corning® Calcein AM Fluorescent Dye at 8 μg/mL concentrations in Hanks Balanced Salt Solution (HBSS) and incubated for 30 min at 37°C, 5% CO_2_. Media were carefully removed from the plates using a Pasteur pipet and plate was washed twice with HBSS. Images were acquired using Zeiss AX10 microscope equipped with a SPOT PURSUIT camera. The tube length, branching length and total area were measured using ImageJ software.

### 4.12 Nitric oxide assays

500 mL of ECs conditioned media was collected and were centrifuged at maximum speed for 5 min and filtered through 10,000 Molecular Weight Cut Off (MWCO) polysulfone filters (Corning Spin‐X UF 500, 431,478). Total nitric oxide and nitrite were measured using colorimetric Arbor Assays following the manufacturer's recommendations. Absorbance was measured at 540 nm using a 96‐well microplate reader. Intracellular nitric oxide levels were measured, using DAF‐FM™ (Thermo Scientific). Endothelial cells were incubated with 10 μM diluted DAF‐FM™ diacetate for 30 min at 37°C. Cells were washed and media was replaced with fresh medium and incubated for an additional 15 min prior to imaging. Images were taken using Zeiss AX10 microscope equipped with a SPOT PURSUIT camera. The intensity of the DAF‐FM fluorescent signal (intracellular NO level) was measured using flow cytometry FACS CantoII (BD), and the data were analyzed by FlowJo sofware.

### 4.13 Cell death assay

PI‐annexin V apoptosis assay was performed according to the manufacturer's instruction (Thermo Fisher, A35122). In brief, 100,000 cells were harvested and rinsed with PBS twice and then resuspended and stained with 100 μL of 1 × annexin V binding buffer, containing 5 μL of annexin V and 5 μL of PI, for 15 min in the dark at room temperature. Stained samples were analyzed by FACS CantoII (BD), and the data were processed by FlowJo software.

### 4.14 Senescence associated β‐galactosidase activity

The fluorogenic substrate C_12_FDG (5‐Dodecanoylaminofluorescein Di‐β‐D‐Galactopyranoside) Invitrogen™ was used to measure β‐galactosidase activity by flow cytometry. Confluent monolayers of muscle cells were incubated with culture media with 33 mM C_12_FDG for 1 hr. at 37°C in the incubator. After staining, the staining solution was removed and the monolayers were rinsed twice with PBS. SMCs were Harvested by trypsinization followed by centrifugation at 1500 rpm for 5 min. Cells were resuspended in ice‐cold PBS at a concentration of ~1 × 10^6^ cells. Samples were analyzed by FACS Canto II (BD) for C_12_FDG fluorescence. The C_12_‐fluorescein signal was measured and β‐galactosidase activity was estimated using the mean of fluorescence intensity (MFI) of the population.

### 4.15 Virus generation and viral infection

Lentiviral constructs expressing GFP‐lamin A, GFP‐progerin, GFP‐control were made as previously described (Kageyama et al., 2014). HEK293T cells were co‐transfected with lentiviral plasmids and 2 virus packaging vectors, pHR‐CMV‐8.2DR and pCMV‐VSVG, utilizing Fugene 6 (Promega). Culture supernatants were collected on 48 hrs and 72 hrs post‐transfection and filtered through 0.45‐μm filters to remove any nonadherent cells. Supernatants were aliquoted and stored at −80 C. Next, HUVEC cells were infected by lentiviruses in media supplemented with Polybrene (Santa Cruze Biotechnology) with the final concentration of 8 mg/mL. The medium was changed every other day post‐infection until the cells were harvested.

The ABE lentivirus construct was generated as described in Ref. Koblan et al. (Koblan et al., 2021). 100,000 iPSCs were transduced with 0.2% v/v concentrated ABE 7.10max lentivirus diluted in mTeSR media for 24 h. The lentivirus was removed, and the media was refreshed next day. Transduced cells were selected after 3 days using 1 μg/mL puromycin. IPSCs were maintained in selection media for 20 days prior to harvesting genomic DNA for editing analysis.

### 4.16 Data analysis

Statistical analyses were performed using GraphPad Prism 7 sofware. Data were evaluated using unpaired Student's t‐test for two groups and one‐way and two‐way analysis of variance (ANOVA) followed by a post hoc Tukey test to compare means of three or more groups. All experiments were repeated at least three times, and the results are presented as the mean ± SEM. A *p*‐value <0.05 was considered significant.

## AUTHOR CONTRIBUTIONS

S.V. and K.C. designed the project. S.V., E.I., H.X., W.A.C., U.L.T., and L.L. performed the experiments. K.S. and G.T. provided ABE‐edited iPSC cells. M.E. and F.C. supported the mouse study, and S.V. and K.C. wrote and edited the manuscript with co‐author contributions. All authors reviewed the manuscript and provided critical analysis.

## CONFLICT OF INTEREST STATEMENT

There are no conflicts of interest.

## Supporting information


Data S1.

## Data Availability

The authors confirm that the data supporting the findings of this study are available within the article and its supplementary materials.
