# Supplementary material for: Angiopoietin‐2 reverses endothelial cell dysfunction in progeria vasculature
Source: Aging Cell. 2024 Oct 18;24(2):e14375. doi: 10.1111/acel.14375 (PMC11822663; doi:10.1111/acel.14375)
Supplement: Supplementary file 1 — Data S1. [file ACEL-24-e14375-s001.docx]

**Supplementary Information**

**Angiopoietin-2 reverses endothelial cell dysfunction in progeria vasculature**

Sahar Vakili^1^, Elizabeth K. Izydore^1^, Leonhard Losert^1^, Wayne A. Cabral^2^, Urraca L. Tavarez^2^, Kevin Shores^3^, Huijing Xue^1,4^, Michael R. Erdos^2^, George A. Truskey^3^, Francis S. Collins ^2^, and Kan Cao^1*^

^1^ Department of Cell Biology and Molecular Genetics, University of Maryland, College Park, MD, USA.

^2^ Molecular Genetics Section, Center for Precision Health Research, National Human Genome Research Institute, National Institutes of Health, Bethesda, MD, USA

^3^ Department of Biomedical Engineering, Duke University, Durham, NC, USA

^4^ Current Address: Frederick National Laboratory for Cancer Research, Frederick, MD, USA

^*^ **Correspondence**
Kan Cao, Department of Cell Biology and Molecular Genetics, University of Maryland, College Park, MD, USA.
Email:

[kcao@umd.edu](mailto:kcao@umd.edu)

**Figure S1**

A) RT-QPCR analysis of P16 and P21in HGPS ECs treated with EC fresh media (FM), HGPS ECs conditioned media (CM, in red) and control ECs conditioned media (CM, in green)

B) Array Map showing the position of each cytokine and controls on a 96-well plate.

C, D) Semi-quantitative analysis of the spots was measured by densitometry and the mean (n = 2 spots) is presented in the graphs as a fold increase or decrease over control. The dashed line indicates a 1.5-fold change cutoff.

E) Western blotting analysis with indicated antibodies on the lysates of control ECs and two HGPS patient ECs.

F) Quantification of fold-change for western blot band densitometry of Ang2 level normalized to control ECs.

G) Representative images of fixed aortas immunostained for human Lamin A/C (green), Ang2 (red), and nuclei (DAPI, blue) in wild-type C57BL mouse at 6 months (i, ii, iii, iv, v, vi) and G608G progeria mouse (vii, viii, viiii, ix, x) at 5x magnification (scale bar = 200 μm).

(H) Representative images of (G) at 40x magnification (scale bar = 100 μm). Autofluorescent elastin fibers in the tunica media appear as wavy lines. Immunofluorescence images were captured with a medium-level exposure time (20 ms).

**Figure S2**

A) Matrigel‐based tube formation assay to assess the angiogenic activity of control and HGPS ECs (scale bars = 200 μm).

B) Quantification of total branching length, tube length, and meshes area for *n*=10 fields of view. Data are presented as mean ± SEM, ***p* < 0.01, *****p* < 0.0001; *n*, 3 independent experiments.

**Figure S3**

A) Representative images from *in vitro* scratch wound healing assays demonstrating ECs cell migration into the cell-free region (outlined) at indicated time points.

B) Summary graph showing typical wound healing (migration) rates by HGPS ECs in the presence of 50 ng/mL of Ang2 vs HGPS ECs.

C) RT-QPCR analysis of indicated genes in HGPS ECs 72 h after treatment with (0, 50, 200, 400 ng/mL) Ang2. Data are presented as mean ± SEM, **p* < 0.05; ***p* < 0.01, ****p* < 0.001, *****p* < 0.0001; *n*, 3 independent experiments.

**Figure S4**

A) Fluorescence images of NO, measured by DAF‐FM staining of control and HGPS ECs treated with (0, 50, 200, 400 ng/mL) Ang2 (scale bars = 50 μm).

B) Quantification of the extracellular total nitric oxide, C) nitrite, and D) nitrate in the conditioned media collected from control and HGPS ECs treated with (0, 50, 200, 400 ng/mL) Ang2. Data are presented as mean ± SEM, ***p* < 0.01, ****p* < 0.001, *****p* < 0.0001; *n*, 3 independent experiments.

**Figure S5**

1. Representative images of HUVECs transduced with GFP‐control, GFP‐lamin A, or GFP‐progerin lentiviral vectors after 48 h (scale bars = 200 μm).
2. Western blotting analysis with indicated antibodies on the lysates of N (not transduced), G (green fluorescent protein), L (Lamin A-GFP), P (progerin-GFP) transduced HUVECs (human umbilical vein endothelial cells).
3. Matrigel‐based tube formation assay to assess vascular network formation activity of HUVECs transduced (CTRL), transduced with GFP‐control, GFP‐lamin A, or GFP‐progerin lentiviral vectors after 18 h (scale bars =200 μm).
4. Quantification of total tube length per field.
5. Quantification of total branching length per field. Data are presented as mean ± SEM, **p* < 0.05; ***p* < 0.01, ****p* < 0.001; *n*, 3 independent experiments.

**Figure S6**

A) Western blotting analysis with indicated antibodies on the lysates of HGPS ECs treated with (0, 50, 200, 400ng/mL) of Ang2 for 24 h.

B) Quantification of fold-change for western blot band densitometry of relative phosphorylated Tyr992 level normalized to corresponding total Tie2.

C) Quantification of fold-change for western blot band densitometry of relative phosphorylated Ser473 AKT level normalized to corresponding total AKT.
